# Interferon-gamma producing CD4^+^ T cells quantified by flow cytometry as early markers for *Mycobacterium avium* ssp. *paratuberculosis* infection in cattle

**DOI:** 10.1186/s13567-024-01324-8

**Published:** 2024-05-31

**Authors:** Hakan Bulun, Philip S. Bridger, Simone Schillinger, Ömer Akineden, Stefanie A. Barth, Marta Fischer, Manfred Henrich, Torsten Seeger, Klaus Doll, Michael Bülte, Rolf Bauerfeind, Christian Menge

**Affiliations:** 1 Institute for Hygiene & Infectious Diseases of Animals, Justus Liebig University Giessen, Giessen, Germany; 2 Institute for Veterinary Food Sciences, Justus Liebig University Giessen, Giessen, Germany; 3 Department for Veterinary Pathology, Justus Liebig University Giessen, Giessen, Germany; 4 Clinic for Ruminants & Swine, University of Giessen, Giessen, Germany; 5 Friedrich-Loeffler-Institut, Institute of Molecular Pathogenesis, Jena, Germany

**Keywords:** Johne’s disease, *M. avium* ssp. *paratuberculosis*, T cell, IFN-γ, flow cytometry

## Abstract

Current diagnostic methods for Johne’s disease in cattle allow reliable detection of infections with *Mycobacterium avium* ssp. *paratuberculosis* (MAP) not before animals are 2 years of age. Applying a flow cytometry-based approach (FCA) to quantify a MAP-specific interferon-gamma (IFN-γ) response in T cell subsets, the present study sought to monitor the kinetics of the cell-mediated immune response in experimentally infected calves. Six MAP-negative calves and six calves, orally inoculated with MAP at 10 days of age, were sampled every 4 weeks for 52 weeks post-inoculation (wpi). Peripheral blood mononuclear cells (PBMC) were stimulated with either purified protein derivatives (PPD) or whole cell sonicates derived from MAP (WCSj), *M. avium* ssp. *avium* or *M. phlei* for 6 days followed by labeling of intracellular IFN-γ in CD4^+^ and CD8^+^ T cells. No antigen-specific IFN-γ production was detectable in CD8^+^ cells throughout and the responses of CD4^+^ cells of MAP-infected and control calves were similar up to 12 wpi. However, the mean fluorescence intensity (MFI) for the detection of IFN-γ in CD4^+^ cells after WCSj antigen stimulation allowed for a differentiation of animal groups from 16 wpi onwards. This approach had a superior sensitivity (87.8%) and specificity (86.8%) to detect infected animals from 16 wpi onwards, i.e., in an early infection stage, as compared to the IFN-γ release assay (IGRA). Quantification of specific IFN-γ production at the level of individual CD4^+^ cells may serve, therefore, as a valuable tool to identify MAP-infected juvenile cattle.

**Supplementary Information:**

The online version contains supplementary material available at 10.1186/s13567-024-01324-8.

## Introduction

Paratuberculosis (Ptb) or Johne’s disease (JD) is an economically significant, incurable, chronic enteric disease of ruminants caused by *Mycobacterium* (*M.*) *avium* subspecies (ssp.) *paratuberculosis* (MAP). Reported herd-level prevalence of Ptb in dairy cattle exceeds 40% in Belgium, Canada, Denmark, France, Germany, Italy, New Zealand, Spain, United Kingdom and the USA [1]. Diagnosis and control are hampered by a considerable long incubation period with only intermittent shedding of the agent and by shortcomings of available test systems for early-stage diagnosis [2, 3]. Calves are most susceptible to MAP infection [4], transmitted either in utero or because neonates ingest milk, colostrum, feed, or water contaminated with faecal material from MAP-shedding adult cattle. In any instance, clinical disease usually does not appear earlier than 2–5 years of age [5–7].

During the long period in which faecal shedding of MAP has not yet started and signs of disease have not yet become apparent, MAP-specific antibodies can only occasionally be detected in infected calves less than 12 months of age even with sensitive serological assays [8]. However, an adaptive cell-mediated immune response (CMI) of the infected host controls the facultative intracellular pathogen during early infection stages [9, 10] with interferon gamma (IFN-γ) as key effector cytokine [10]. Activated CD4^+^ T cells are the main source of IFN-γ even though it is also produced by CD8^+^ and γδ T cells [11–13]. CD8^+^ T cells play an effective role by preventing γδ T cell-mediated destruction or by inactivating MAP antigen-activated CD4^+^ T cells [12, 14]. In turn, γδ T lymphocytes influence the function of monocytes during MAP infection [15]. Unfortunately, assays to detect antigen-specific CMI by means of purified protein derivative (PPD)-induced IFN-γ release in whole blood samples (IFN-γ release assay, IGRA) often give rise to false-positive and inconclusive results [16]. This relates to the lack of discrimination between IFN-γ producing antigen-specific T lymphocytes (i.e., CD4^+^ and CD8αβ^+^ T cells) and non-specific IFN-γ production by innate immune cells (i.e., γδ T cells and natural killer cells) in response to exposure to mycobacterial molecular patterns [17, 18]. Immunophenotyping of peripheral blood mononuclear cells (PBMC) by flow cytometry analysis (FCA) allows to differentiate T cell subsets and to detect cytokines at the subpopulation level. Ex vivo/in vitro induced, antigen-specific IFN-γ production by PBMC was suggested as prime read-out when testing animals early after natural and experimental MAP infections [19–22]. FCA-based quantification of IFN-γ in CD4^+^ lymphocytes at single cell level proved to be superior in identifying antigen-specific T cells in experimentally MAP infected cattle less than 12 month of age, i.e., 40 weeks post-infection [19]. However, the test was conducted at one timepoint post-infection only and it remains to be determined how this response develops over time to identify the earliest timepoint at which the method may be of higher diagnostic value as the IGRA.

MAP-specific antigens are essential for the sensitive, specific and early detection of the infection [23–25]. Most of the reported MAP antigens, however, are shared with other mycobacteria, and MAP shows about 99% DNA homology with the widespread *M. avium* subspecies *avium* [24, 26]. Recombinant antigens have been evaluated for use in diagnostic tests with various success [14, 17, 18, 25, 27–35]. Purified protein derivatives (PPDs) are the most commonly used antigens for testing CMI to mycobacterial infections [36], but whole cell sonicate (WCS) antigen preparations have also been evaluated [19, 37]. WCS from MAP (WCSj) provided more specific test results in comparison to a cocktail of MAP recombinant antigens [30, 38–41], whereas PPD preparations derived from *M. avium* ssp. *avium* (PPDa) and from MAP (PPDj) were more potent than a WCS antigen from MAP (WCSj) at stimulating IFN-γ in another study [42].

Goat models of Ptb [43, 44] that are less resources-demanding than bovine models, became increasingly popular for testing vaccine efficacies [45, 46] and for the search for better diagnostic antigens [34]. The longitudinal study presented here was conducted with young bovines to account for cattle-specific features in the MAP-induced immune response. Aiming to detect MAP infection at the earliest time point possible, the dynamics of antigen-specific CMI was tightly monitored in experimentally infected calves. In vitro stimulation of PBMC was combined with FCA [19] as read-out to distinguish between innate and adaptive immune cell responses in young animals. Data were interpreted also in comparison to IGRA results. Different MAP antigen mixtures were used for stimulation to assure high sensitivity. To compensate for possible loss of specificity, data were compared and normalized to those obtained with preparations from other mycobacterial species.

## Materials and methods

### Identification of MAP-negative and MAP-positive herds and sampling for the pilot study

To identify suitable sources of calves for the longitudinal study, eight dairy cattle herds were screened for the absence of MAP-infected animals. Herds were selected based on anamnestic data (breed: German Holstein; no recent reports of clinical paratuberculosis; closed herds; cooperative farmer) [8]. Initially, all cattle aged ≥ 24 months were tested serologically using two commercial ELISA test kits (Svanovir^®^ Para-TB-Ab-ELISA [Svanovir^®^] and Pourquier^®^ ELISA-Paratuberculosis screening [Pourquier^®^]) applying a two-step sampling and analysis approach as described by Bottcher and Gangl [47]. If the herds were considered serologically negative (no Pourquier^®^-positive animals), faecal samples of all animals aged ≥ 24 months were tested by PCR and culture [48]. Four MAP-negative herds were identified, consisting of 37 to 65 cows ≥ 24 months. Classification was confirmed by a second screen (serological and faecal examinations) performed 12–18 months after the first one.

To obtain positive control samples, animals aged ≥ 24 months from another cattle herd (Herd 5) with a history of clinical paratuberculosis were sampled (faeces and serum) and tested for their MAP-status.

From the tested herds, five MAP-negative cows (Herd 3) and seven MAP-infected animals (Herd 5) were selected for a pilot study (Additional file 1) and blood samples drawn (see below) to develop the draft FCA protocol.

### Longitudinal study design

Twelve female German Holstein calves were taken from the MAP-negative herds at the age of 4–7 days post-natum (pn). Animals were housed in isolated animal rooms. Until the age of 60 days pn, calves were held separate in straw-bedded boxes with no direct contact to one another. Thereafter, they were kept in pairs in pens in isolated rooms. As soon as the youngest calf of one run (see below) was 6 months old, the entire group (*n* = 6) was moved to a straw-bedded freestall barn and kept there until necropsy. The longitudinal study was conducted in two staggered runs with initially six noninfected calves (E1-6) as the control group and six calves (E7-12) as the MAP-infected group. After moving calves of the control group to the freestall barn, the pens and rooms were intensively cleaned, disinfected and then left empty for 8 weeks. Approximately 8 months after the study had started with the control group, calves of the MAP-infected group were moved into these rooms. Each run was terminated after 52 weeks post-inoculation (wpi). None of the calves showed clinical signs of paratuberculosis throughout the entire study period but some MAP-unrelated medical conditions required veterinary intervention. Calf E9 got a severe peritonitis and, after unsuccessful medical treatment, had to be euthanized at the age of 253 days. This calf was sampled up to 34 wpi only (Table 2). Calf E2 developed a mechanical ileus in the 39^th^ wpi which was successfully resolved via surgery. Calf E4 experienced a rotavirus infection in the 3^rd^ wpi and was successfully treated by oral rehydration therapy. Calf E5 received iron supplementation in the 1^st^ week of life after iron deficiency was diagnosed. Calf E6 developed a left abomasal displacement in the 10^th^ and 11^th^ wpi which could be resolved without surgery by rolling technique.

### MAP inoculation procedure

Following the suggestion of an international expert group [49], oral inoculation via milk replacer deploying MAP strain K10 was conducted to most closely mimic the natural route of the infection. On days 10, 12, and 14 pn calves of the MAP-infected group were orally challenged with an amount of 3.5 × 10^8^ colony-forming units (CFU) per inoculum (1.05 × 10^9^ CFU in total) of MAP strain K10 (ATCC^®^ BAA-968, purchased via LGC Promochem GmbH, Wesel, Germany). To prepare the inocula, the bacteria were incubated in Middlebrook 7H9 medium supplemented with 2 mg of mycobactin J, 10% oleic acid-albumin dextrose catalase (OADC) growth supplement (Becton Dickinson [BD], Heidelberg, Germany) and 0.05% Tween^®^ 80, for 4–8 weeks (at 37 °C) until the optical density (OD_660 nm_) was 0.3. Quality of the inocula was assured by culturing a sample on blood agar plates to exclude contaminations as well as by Ziehl–Neelsen staining, PCR for gene *251* [50], nested PCR for IS*900* [51] and real-time duplex PCR for F*57* and IS*Mav*2 [52, 53]. Numbers of viable bacteria per milliliter was determined with the LIVE/DEAD^®^ BacLight™ kit (Invitrogen GmbH, Karlsruhe, Germany) and by solid agar counts. Aliquots of 5 mL suspensions containing 3.5 × 10^8^ CFU MAP strain K10 were stored at −70 °C. Twenty-four hours prior to inoculation, aliquots were diluted in 20 mL supplemented Middlebrook 7H9 medium, kept at 37 °C overnight and then fed orally, suspended in one liter of milk replacer per calf. The calves of the control group received mock inocula (supplemented Middlebrook 7H9 medium without bacteria).

### Sampling

The pre-challenge samples (faeces, serum, and whole blood samples) were taken one day before the first inoculation of MAP bacteria. In the first 2 weeks after the inoculations, faecal samples were taken with 2 days intervals, then on a weekly basis for further 2 weeks and finally every 2 weeks until the end of the study. Whole blood samples were taken every 4 weeks, starting day 9 pn until 2 days prior to euthanasia (14 samplings in total). Besides faecal, serum and whole blood samples, intestinal lymph node biopsies were taken at 2, 4, 13, 24 wpi by minimally invasive laparoscopic surgery and on the day of the euthanasia as part of the necropsy procedure. After 12 months of observation, calves were euthanized and necropsied including extensive sampling of various tissues of different locations (data not shown).

Serum samples were collected monthly during the observation period and tested for antibodies against *M. avium* subsp. *paratuberculosis* by ELISA and a novel FC based assay [8]. MAP-specific antibodies could only occasionally be detected in the infected calves.

Whole blood samples for both, IFN-γ release assay (IGRA) and IFN-γ flow cytometry analysis (FCA) were taken via puncture of the *Vena* (*V*.) *jugularis externa* after disinfection of the puncture site with 75% ethanol. An indwelling cannula (18 G (1.3 × 45 mm), Braun^™^ Melsungen, Germany) was attached to an elongation set (150 cm; Santec^™^, Heimbuchenthal, Germany) and the blood drawn into 60 mL syringes prefilled with a 3.8% citrate solution (0.2 mL per 1 mL blood). Whole blood samples for IGRA were drawn into plasma collection tubes treated with lithium heparin (5 mL; Kabe, Nümbrecht-Elsenroth, Germany). All samples were kept at room temperature (RT) until further processing after 5 h (for IGRA) and after 10 min to 1.5 h (for IFN-γ FCA).

### Antigens for in vitro stimulation

Purified protein derivatives (PPD) derived from *M. avium* ssp. *avium* (PPDa), *M. bovis* (PPDb), and MAP (PPDj) were obtained from external sources (Table 1). PPD derived from *M. phlei* (PPDp) was produced as described in the Manual of Diagnostic Tests and Vaccines for Terrestrial Animals [54]. Briefly, *M. phlei* bacteria (DSM No. 43239; German Collection of Microorganisms and Cell Cultures GmbH, Braunschweig, Germany) were cultured in 700 mL Middlebrook 7H9 medium (pH 7.3 ± 0.2; 37 °C, 10–12 days) until a pellicle (1–2 mm) was formed on the surface of the liquid medium, then autoclaved (30 min, 121 °C). After filtration with folded filter (Ø 70 mm), the filtrate was precipitated by slowly adding 40% trichloroacetic acid (TCA; 9 parts filtrate and 1 part 40% TCA; final concentration of TCA was 4%) on an agitator. The precipitate was allowed to settle at RT overnight, pelleted and washed twice in 50 mL 1% TCA (15 min, 2500 × *g*), and resuspended in 50 mL 5% NaCl solution (pH adjusted to 2.7 by adding 32% HCl solution). After centrifugation (15 min, 2500 × *g*), precipitate was dissolved in 10 mL alkaline solvent (17.2 g Na_2_HPO_4_ x H_2_0, 0.9 g NaOH ad 1 L; pH 6.6) and centrifuged to remove insoluble material. An aliquot was taken to determine the protein concentration (Pierce™ BCA Protein Assay Kit, Thermo Fisher, Germany). Finally, an amount of 50 µL phenol and 10 mL glucose buffer was added per 10 mL preconcentrate and sterile filtrated using a syringe filter (pore size 0.45 µm). The resulting solution (PPDp) was aliquoted and stored at 4 °C until further use.

**Table 1:** **Antigen preparations used to stimulate blood cell cultures**

Antigen^1^	Mycobacterial species	Final protein concentration used in culture	Application
PPDb^2^	*M. bovis*	15 µg/mL	IGRA
PPDa^2^	*M. avium* ssp. *avium*	15 µg/mL/5 µg/mL	IGRA/IFN-γ FCA
PPDj^3^	*M. avium* ssp. *paratuberculosis*	5 µg/mL	IFN-γ FCA
PPDp	*M. phlei*	5 µg/mL	IFN-γ FCA
WCSa	*M. avium* ssp. *avium*	1.5 µg/mL	IFN-γ FCA
WCSj^4^	*M. avium* ssp. *paratuberculosis*	1.5 µg/mL	IFN-γ FCA
WCSp	*M. phlei*	1.5 µg/mL	IFN-γ FCA

^1^PPD: Purified protein derivative, WCS: Whole cell sonicate.

^2^Prionics, Planegg-Martinsried, Germany.

^3^Kindly provided by Ch. Thoen, Department of veterinary microbiology and preventive medicine, College of veterinary medicine, Iowa state university, Ames IA, USA.

^4^Kindly provided by R. W. Waters, bacterial diseases of livestock unit, national animal disease center, ARS, USDA, Ames IA, USA.

WCS of MAP strain K10 was produced according to Waters, Miller, Palmer, Stabel, Jones, Koistinen, Steadham, Hamilton, Davis and Bannantine [19] (Table 1). The same method was used in our laboratory to prepare WCS derived from *M. avium* ssp. *avium* (DSM 44156) and *M. phlei* (DSM 43239). To this end, the mycobacterial organisms were cultured (500 mL Middlebrook 7H9 medium, 37 °C) to an OD_660 nm_ of 0.2 to 0.9. Mycobacteria were pelleted (3000 × *g*, 4 °C, 30 min), the pellet was washed twice (40 mL cold PBS, 3000 × *g*, 4 °C, 30 min) and resuspended in 10 mL PBS by sonication on ice with a probe sonicator (Branson Sonifier Cell Disruptor B15, Germany; three cycles 30 s, output control 4–5 hold, continuous, 40% duty cycle). The suspension was transferred into 1 mL FastPrep tubes containing ceramic beads (Ø 1.4–1.6 mm, 1.2–1.4 g) followed by homogenization twice (Precellys 24, Peqlab Biotechnologie GmbH, Erlangen, Germany; 45 s, 65 movement/s, 5 min chilling periods (on ice) between both steps). Upon centrifugation (13 000 rpm, 5 min), supernatants were harvested and filtrated through 0.45 µm and 0.22 µm filters, consecutively. After determining the protein concentration (BCA^™^ Protein Assay Kit), 200 µL aliquots were stored at −70 °C until further use.

### IFN-γ release assay (IGRA)

A commercially available test kit (BOVIGAM^®^; Prionics, Planegg-Martinsried, Germany) was applied according to the instructions by the manufacturer with an incubation period of blood samples in the presence of antigen of 16–24 h. However, data were processed differently. Due to the close relationship of *M. avium* ssp. *avium* to MAP, PPDa cross-reacts with (i.e., activates) MAP-specific T cells [55]. Therefore, PPDa was used as target antigen and PPDb as negative control antigen in this study. Accordingly, values measured by ELISA-reader using a 450 nm filter and a 620 nm reference filter were used for calculations as follows: X_1_ = PPDa [Absorbance]_620–450 nm_–Nil [Absorbance]_620–450 nm_; X_2_ = PPDa [Absorbance]_620–450 nm_–PPDb [Absorbance]_620–450 nm_. Nil values were obtained with blood samples incubated in the absence of antigen (PBS only), i.e. containing spontaneously released IFN-γ only. Test results were interpreted analogously to the contemporary recommendations of the manufacturer (package insert, version 1.3e) as negative (X_1_ < 0.1 and X_2_ < 0.1), positive (X_1_ > 0.1 and X_2_ > 0.1), and inconclusive (X_1_ > 0.1 and X_2_ < 0.1 or X_1_ < 0.1 and X_2_ > 0.1).

### IFN-γ flow cytometric assay (FCA)

**Figure 1:**
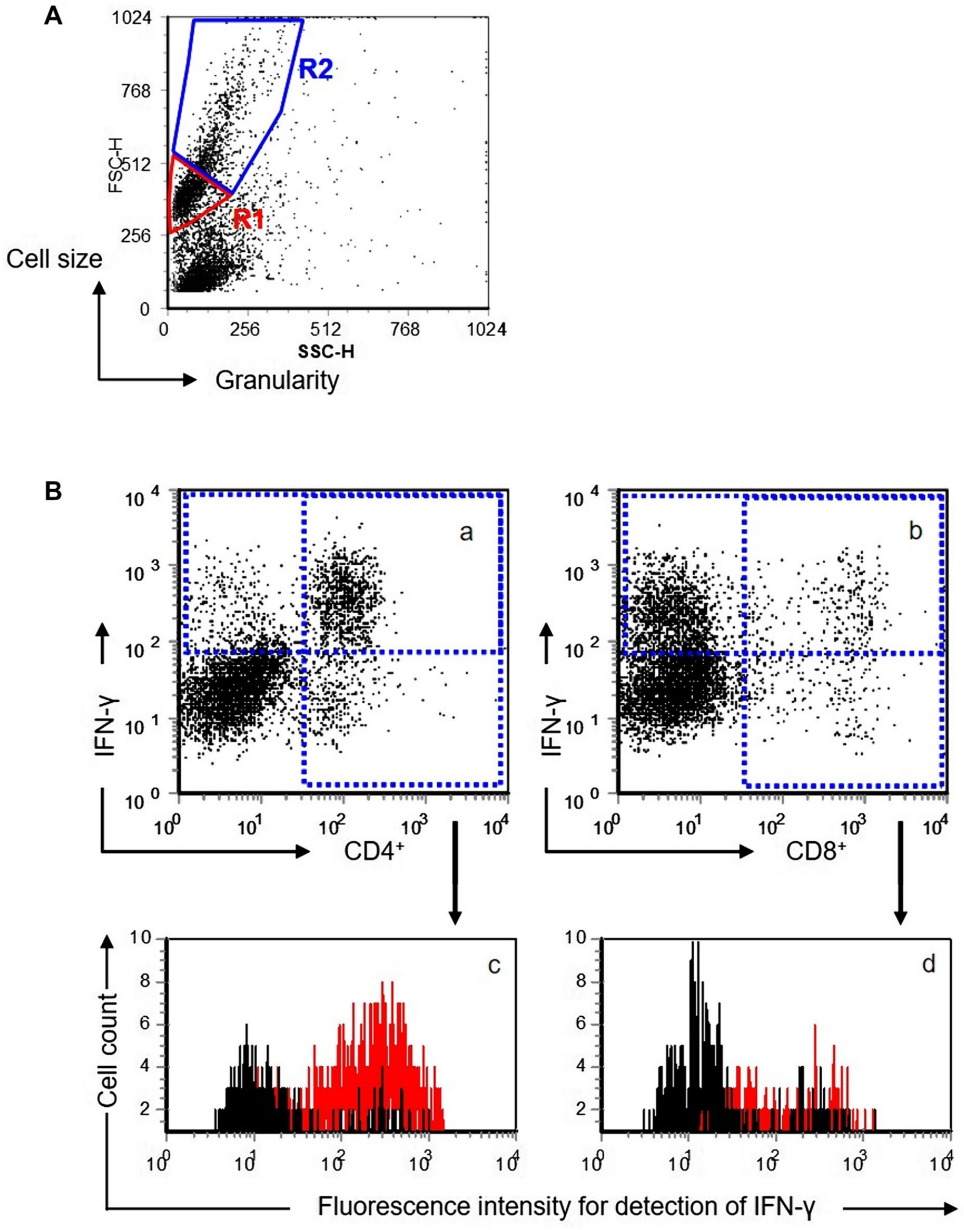
**Strategy to analyse flow cytometrically acquired data.** **A** Representative scatter plot with gates for lymphocytes (R1) and for lymphoblasts (R2). **B** gating strategy to analyse IFN-γ in CD4^+^ and CD8^+^ T cells, respectively. Representative dot plots of gated lymphoblasts to detect IFN-γ^+^, CD4^+^ and CD8^+^ cells, respectively (**a**, **b**). Representative histograms depict the mean fluorescence intensity (MFI) of the IFN-γ signal in CD4^+^ and CD8^+^ lymphoblasts, respectively [c, d]. Black curves represent MFI data of unstimulated (medium control) CD4^+^ (**c**) and CD8^+^ (**d**) lymphoblasts. Red curves represent fluorescence intensity data of WCSj stimulated CD4^+^ (**c**) and CD8^+^ (**d**) lymphoblasts

Bovine PBMC were isolated by Ficoll gradient centrifugation [56, 57] and seeded in 12-well plates in cell culture medium (RPMI 1640 supplemented with 10% fetal calf serum, 3 μM 2-mercaptoethanol, 100 IE/mL penicillin, 2 mM L-glutamine) at 4 × 10^6^ cells/well in 1 mL. Then, 5 µg/mL PPDj, 5 µg/mL PPDa, 5 µg/mL PPDp, 1.5 µg/mL WCSj, 1.5 µg/mL WCSa or 1.5 µg/mL WCSp, respectively, was added (2 wells per condition). Each experiment included two wells of PBMC incubated without any antigens as negative (medium) control. A positive (stimulation) control consisted of two wells of PBMC cultures incubated with 1.5 µg/mL concanavalin A. PBMC-cultures were incubated for 6 days (37 °C, 5% CO_2_, 95% humidity). On the 6^th^ day, PBMC cultures were boosted for 4 h by addition of 1 µg/mL ionomycin and 50 ng/mL phorbol-12-myristate-13-acetate; 10 µg/mL brefeldin A was added to prevent secretion of IFN-γ. Thereafter, PBMC of duplicate cultures per condition were pooled and 180 µL of the resulting suspension was transferred per well to a 96-well V-shaped microtiter plate. Cells from each condition were tested in six samples (secondary antibody control, CD4 / IFN-γ, and CD8/IFN-γ; each in duplicate). After centrifugation (400 × *g*, 3 min, 4 °C), pellets were resuspended in 50 µL of an in-house produced hybridoma cell supernatant containing either anti-bovine CD4 (mouse IgG_2a_, cell line IL-A11) or anti-bovine CD8 (mouse IgG_2a_, IL-A105). After 15 min incubation on ice, cells were centrifuged, washed, and resuspended in 50 µL of biotin conjugated anti-mouse IgG2a (R19-15; BD; diluted in PBS + 1% BSA + 0.01% of sodium azide) and 1 µg/mL of 7-amino actinomycin D (7-AAD). After 15 min on ice, cells were washed once, and resuspended in 50 µL of allophycocyanin (APC)-conjugated streptavidin (Southern Biotech, Birmingham, U.S.A.; diluted in PBS + 1% BSA + 0.01% of sodium azide), left for 15 min on ice, washed once in 100 µL PBS, resuspended in 50 µL of Leucoperm™ reagent A (Serotec, Düsseldorf, Germany; fixation reagent) and incubated. Upon centrifugation and one washing step in PBS, cells were resuspended in 50 µL anti-bovine IFN-γ (CC302, mouse IgG_1_; Serotec) diluted 1:300 in Leucoperm^™^ reagent B (permeabilization reagent; Serotec). Secondary antibody controls were diluted in Leucoperm^™^ reagent B. After a 20 min incubation period, cells were centrifuged and resuspended in 50 µL Leucoperm^™^ reagent B containing R-phycoerythrin (R-PE) -conjugated anti-IgG1 (Southern Biotech). After a 20 min incubation period, cells were centrifuged, washed once in PBS, resuspended in 50 µL PBS and transferred into 5 mL tubes prefilled with 150 µL of PBS for flow cytometric analysis with a FACSCalibur^™^ flow cytometer using the software Cell Quest Pro (BD). A live-gate was set around morphologically intact lymphocytes and lymphoblasts (R1 and R2 in Figure 1A) and the measurements were terminated as soon as 10 000 events were acquired or after a period of 50 s. Data were exported and analyzed with FCS Express 2 software (De Novo-Software, Thornhill, Ontario, Canada). Using a forward-scattered light (FSC) vs. side-scattered light (SSC) plot, both lymphocytes (R1) and lymphoblasts (R2) were gated separately. The gated events were assigned to a FL-4 (CD4/CD8) vs. FL-2 (IFN-γ) dot plot (Figure 1B). Quadrants were set according to the negative control defining less than 2% of the cells as positive, i.e., present in the upper and right quadrants. In order to determine the mean fluorescence intensity (MFI) of the IFN-γ signal within CD4^+^ and CD8^+^ T cells, respectively, both right quadrants (i.e., CD4^+^ and CD8^+^ T cells, respectively) were assigned to a FL-2 histogram. Arithmetic means of duplicate determinations were calculated. Resulting data of antigen responses in both, the control and the MAP-infected groups, were calculated using median, maximum and minimum of the values in each group normalised versus unstimulated cells (medium) following the formula: value [PPD (WCS) antigens]/value [medium]. Alternatively, values obtained with PPDa or PPDj and WCSa or WCSj stimulated cultures were normalised to values obtained with PPDp and WCSp, respectively.

### Statistical analyses

Differences between the incubation conditions in general were calculated by using 2-way Analyses of Variance (ANOVA) for repeated measurements (SPSS Inc.). Significant differences between each incubation condition for each time point were determined by using the Bonferroni test for pairwise comparisons including Greenhouse–Geisser correction and the Student’s *t*-test. Two cut-offs were defined by using the values of control group calves. The 95% and 99% quantile values were used to determine negative, inconclusive, and positive results of MAP-infected calves.

To estimate the sensitivity and specificity of the tests, further analysis was performed by also using corrected OD values for the data obtained by ELISA by calculating cOD_PPDa_ = OD_PPDa_–OD_Nil_; cOD_PPDb_ = OD_PPDb_–OD_Nil_ and the difference of the OD values (diffOD = OD_PPDa_–OD_PPDb_). A comparison between the control calves and the MAP-infected calves over time was visualized in dot plots (Figure 8) to appreciate the distribution of the measured values per group. The sensitivity and specificity were calculated by using a receiver operating characteristic (ROC) curve analysis for only selected incubation conditions. Statistical analyses for these purposes were performed using MedCalc Statistical Software for Windows version 13.1.0 (MedCalc Software bvba, Ostend, Belgium; version 2014).

## Results

### MAP-unexposed and naturally MAP-infected cows (pilot study)

Four out of five cows from a MAP-negative herd, referred to as MAP-unexposed cows from hereon, tested negative by IGRA while results of one animal were interpreted inconclusive (Additional file 2). Blood samples from four out of seven cows which shed MAP bacteria in their faeces and were serologically MAP-positive, referred to as MAP-infected cows from hereon, reacted IGRA positive. One cow was judged IGRA-inconclusive, the remaining two animals were negative.

Blood samples of the animals were also analyzed using the FCA. Different parameters were assessed to identify the best approach to detect a MAP-specific CMI. In a first approach all morphologically intact cells (lymphocytes and lymphoblasts = all-cell-analysis) were analyzed. In the second step, the intact cells were divided into two subgroups, i.e., lymphocytes and lymphoblasts. Analysis of mean fluorescence intensities (MFI) of the IFN-γ signal and the percentage of IFN-γ^+^ cells in lymphocytes and lymphoblasts revealed minor differences between MAP-infected and MAP-unexposed cattle by all-cell-analysis of all cultured cells after incubation with antigens (Additional file 3). The response of lymphoblasts to all antigens tended to be higher in MAP-infected cattle than in MAP-unexposed cattle but data from MAP-infected cattle showed stark variation.

**Figure 2:**
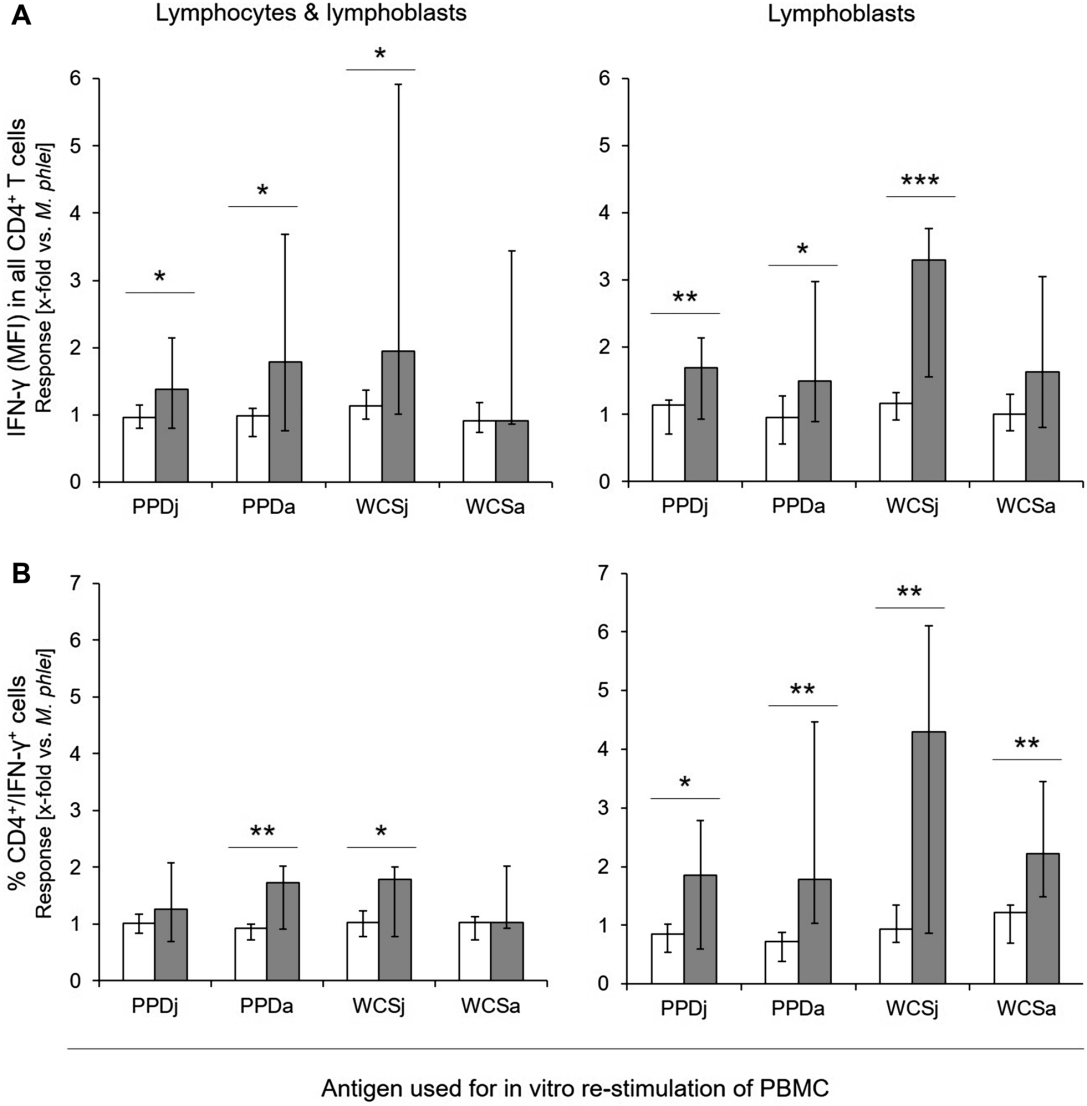
**Relative portion and IFN-γ production by CD4**^**+**^** T cells from MAP-unexposed and MAP-infected cows after in vitro stimulation with mycobacterial antigens.** Data are median (± max / min) of the values with duplicates from MAP-unexposed (*n* = 5) [white bars] and MAP-infected (*n* = 7) animals [grey bars], normalised to values obtained after stimulation with a respective (i.e., PPD and WCS, respectively) *M. phlei* preparation. Asterisks above each condition indicate significant differences between MAP-unexposed and MAP-infected cows [Student’s *t*-test; *p* ≤ 0.001 (***), *p* ≤ 0.01 (**), or *p* ≤ 0.05 (*)]. MFI: Mean fluorescence intensity.

**Figure 3:**
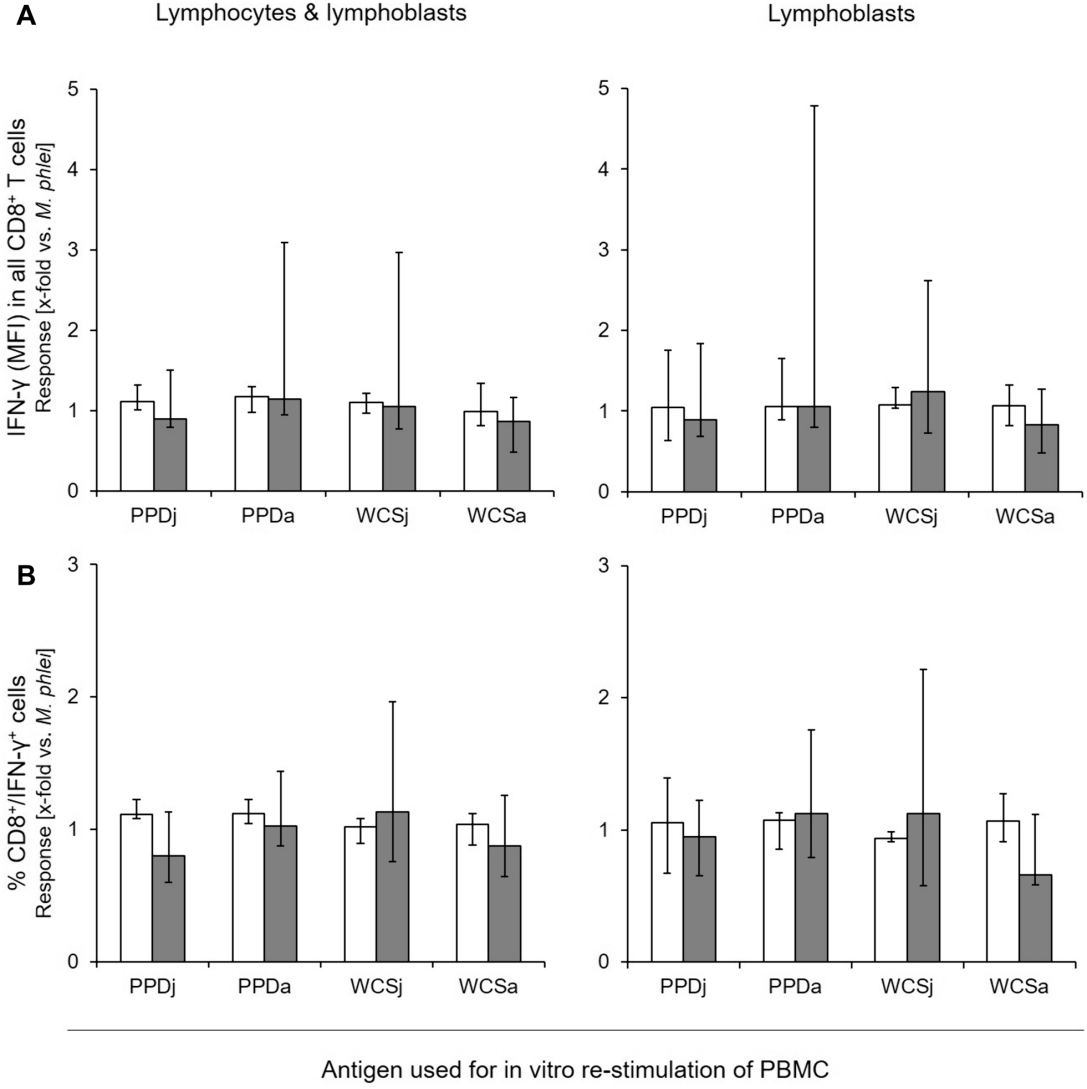
**Relative portion and IFN-γ production by CD8**^**+**^** T cells from MAP-unexposed and MAP-infected cows after in vitro stimulation with mycobacterial antigens.** Data are median (± max / min) of the values with duplicates from MAP-unexposed (*n* = 5) [white bars] and MAP-infected (*n* = 7) cows [grey bars], normalised to values obtained after stimulation with a respective (i.e., PPD and WCS, respectively) *M. phlei* preparation. Asterisks above each condition indicate significant differences between the MAP-negative and the MAP-infected group [Student’s *t*-test; *p* ≤ 0.001 (***), *p* ≤ 0.01 (**), or *p* ≤ 0.05 (*)]. MFI: Mean fluorescence intensity.

IFN-γ production was therefore analyzed at subpopulation level, i.e., by assessment of MFI values for the IFN-γ signal in CD4^+^ and CD8^+^ T cells, respectively, and by assessment of the percentage of double positive cells (CD4^+^/IFN-γ^+^, CD8^+^/IFN-γ^+^). Neither PBMC of MAP-unexposed cows nor PBMC of MAP-infected cows responded to in vitro stimulation with PPDp and WCSp (data not shown). To correct for a possible CMI of cows to environmental mycobacteria, data were normalized to data obtained after in vitro stimulation with *M. phlei* antigen preparations. Applying this algorithm, MFI of the IFN-γ signal in CD4^+^ PBMC stimulated with PPDj, PPDa or WCSj, respectively, was significantly higher in MAP-infected cattle than in MAP-unexposed cows (*p* = 0.037, *p* = 0.049, and *p* = 0.047 respectively; Figure 2A). Differences between MAP-infected and MAP-negative cattle became more prominent if analyses were restricted to the lymphoblast population. Responses of lymphoblasts from MAP-infected cattle to PPDj and PPDa were higher than responses of lymphoblasts from MAP-unexposed cattle (*p* = 0.009 and 0.038, respectively). Difference between the two groups was greatest when lymphoblasts were stimulated with WCSj antigen (*p* < 0.001). Similar results were obtained by analyzing the “percentage of double-positive cells” (% CD4^+^/IFN-γ^+^ cells; Figure 2B), i.e., analysis of lymphoblasts revealed higher values in MAP-infected cows throughout. By contrast, no significant group differences occurred in the reactivity to mycobacterial antigens of CD8^+^ PBMC independent of the analytical approach (Figure 3).

### Experimentally infected calves (longitudinal study) tested by IGRA

IGRA results were not reliable to determine the infection status in the first 12 weeks after inoculation (Table 2). Results of both, the control group and the MAP-infected group, were more consistent between 16–32 weeks post-inoculation (wpi). A total of 60 samples (30 from each group) was tested in that period of time. Altogether, 27 samples from the control group gave negative, two samples gave inconclusive and one sample yielded positive results. On the opposite, eight samples of MAP-infected calves tested negative, five samples were graded inconclusive and 17 samples positive. From 36^th^ week post-inoculation until the end of the experimental studies, IGRA results for whole blood cells were again not consistent.

**Table 2:** **IFN-γ release in whole blood cell cultures from calves stimulated in vitro with mycobacterial antigen (IGRA)**

		Weeks post-inoculation													
	Calf	0	4	8	12	16	20	24	28	32	36	40	44	48	52
Control group	E1	n.t	?	+	−	?	−	−	−	−	−	−	+	−	+
	E2	−	+	?	−	−	−	−	−	+	+	+	+	−	+
	E3	?	+	−	?	?	−	−	−	−	?	?	−	−	−
	E4	−	?	−	−	−	−	−	−	−	+	+	+	−	n.t
	E5	−	?	−	−	−	−	−	−	−	−	−	−	−	−
	E6	?	−	−	+	−	−	−	−	−	−	−	−	−	+
MAP-infected group	E7	−	−	−	?	?	+	−	+	+	−	−	+	+	+
	E8	−	−	−	?	+	−	+	+	+	+	−	?	+	+
	E9	−	−	?	+	+	+	+	+	?	n.t	n.t	n.t	n.t	n.t
	E10	−	−	−	−	?	?	+	?	−	+	+	?	+	+
	E11	−	+	?	+	+	+	+	−	−	−	?	−	−	−
	E12	−	−	−	−	+	+	−	−	−	n.t	?	?	+	+

^*^n.t. (not tested), negative (–), positive ( +), inconclusive (?).

### Comparison of antigens for the FCA

**Figure 4:**
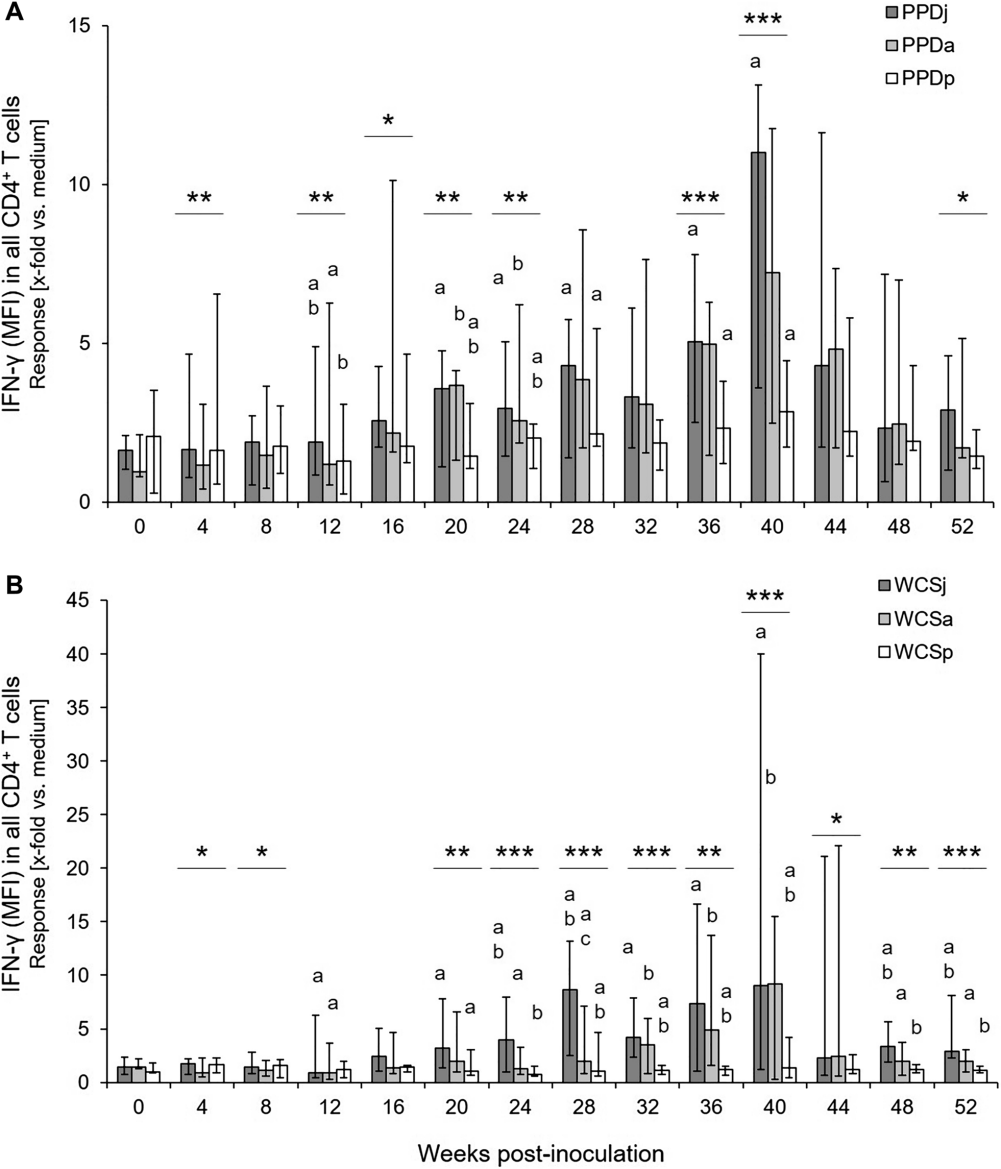
**Antigen dependent CD4**^**+**^** T cell response in vitro of MAP-infected calves over time.** The mean fluorescence intensities (MFI) of the measured cells for the detection of IFN-γ were taken as indicator for the average amount of the cytokine produced by the cells. The reaction titers reflect the x-fold increased MFI for IFN-γ in CD4^+^ cells after incubation with PPDj, PPDa and PPDp [**A**] or WCSj, WCSa and WCSp [**B**] in relation to incubation without antigens (medium control). Bars indicate median (± max/min) values (*n* = 5–6). Asterisks above each time point indicate significant differences between the respective mycobacterial antigens in general [two-way ANOVA for repeated measurements; *p* ≤ 0.001 (***), *p* ≤ 0.01 (**), or *p* ≤ 0.05 (*)]. Same letters above bars indicate significant differences (Bonferroni test for pair wise comparisons; *p* < 0.05).

Based on the findings with adult animals, quantitation of MFI for the detection of IFN-γ in CD4^+^ T cells was applied to antigen stimulated PBMC of experimentally MAP-infected calves. Different from adult animals, lymphocytes and lymphoblasts were analyzed together as the numbers of CD4^+^ lymphoblasts was found to be very low in juvenile cattle. For ease of reading, cells analyzed from blood samples of experimentally infected and control calves are referred to as “CD4^+^ T cells” below. When the MFI of the IFN-γ signal in CD4^+^ T cells was used to compare PBMC responses to MAP, *M. avium* ssp. *avium* and *M. phlei* antigens within the group of inoculated animals, a general difference between PPD antigens was detected throughout the study period (Figure 4A). Increase in production of IFN-γ was particularly significant in PPDj and PPDa stimulated CD4^+^ T cells in comparison to PPDp at 20 wpi. Response to PPDj antigen also was significantly higher than the response to PPDp antigen at 28, 36, and 40 wpi. Also, a general difference in the responsiveness to WCS antigens was detected at 4 and 8 wpi, and from 20 wpi onwards until the end of the study period (Figure 4B). No antigen-specific IFN-γ production was detectable in CD8^+^ cells throughout (data not shown).

### Comparison of MAP-infected and control calves in the FCA

**Figure 5:**
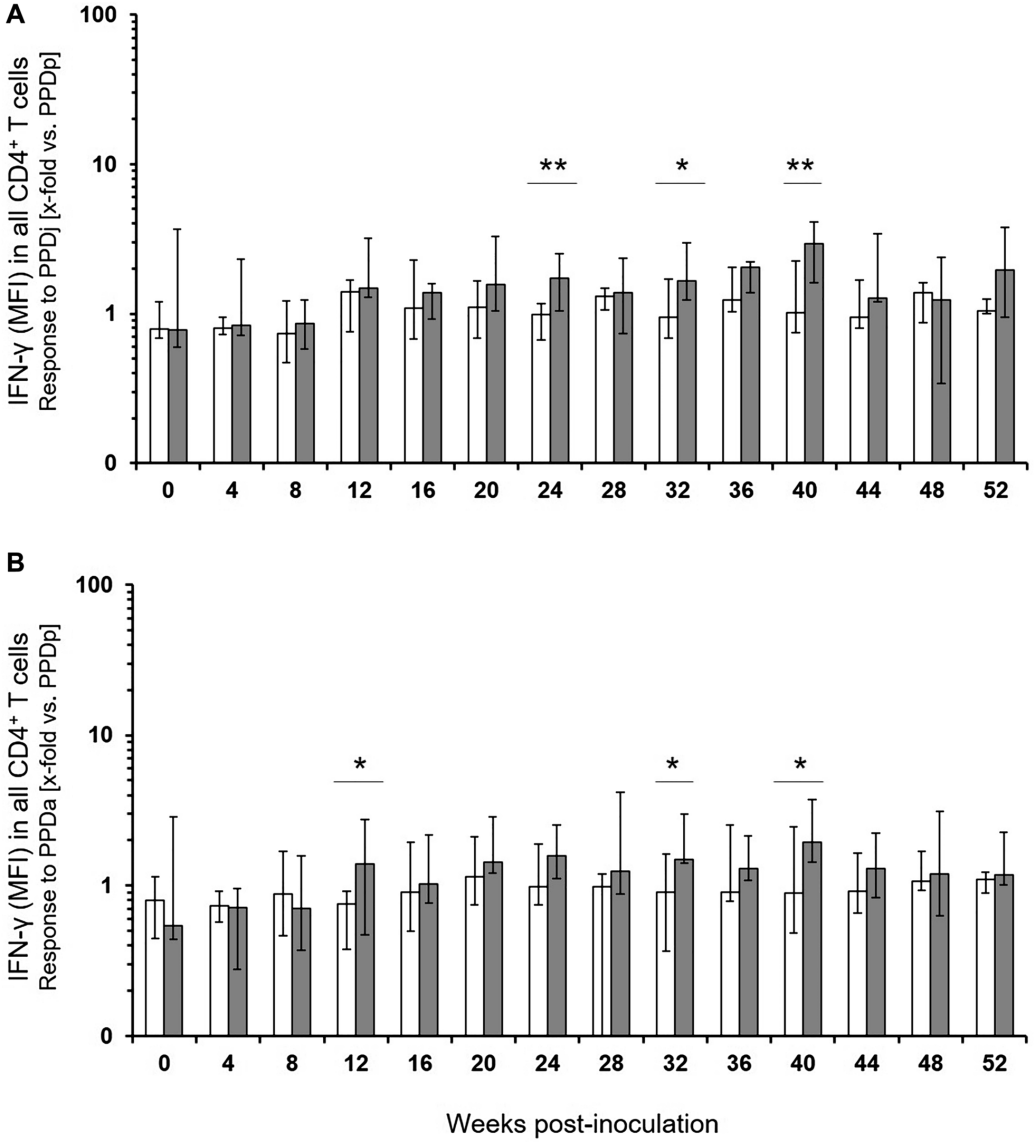
**Mean fluorescence intensities of the IFN-γ signal in CD4**^**+**^** T cells of control group and MAP-infected calves after stimulation with mycobacterial antigens in vitro.** The mean fluorescence intensities (MFI) of the measured cells for the detection of IFN-γ were taken as indicator for the average amount of the cytokine produced by the cells. Cells were stimulated with PPDj (**A**) and PPDa (**B**). Data were normalised to values obtained after PPDp stimulation and presented as median (± max / min) of the values with duplicates from MAP-negative [*n* = 6; white bars] and MAP-infected animals [*n* = 5–6; dark-grey bars]. Significant differences between animal groups were determined by Student’s *t*-test and depicted if *p* ≤ 0.01 (**), or *p* ≤ 0.05 (*).

**Figure 6:**
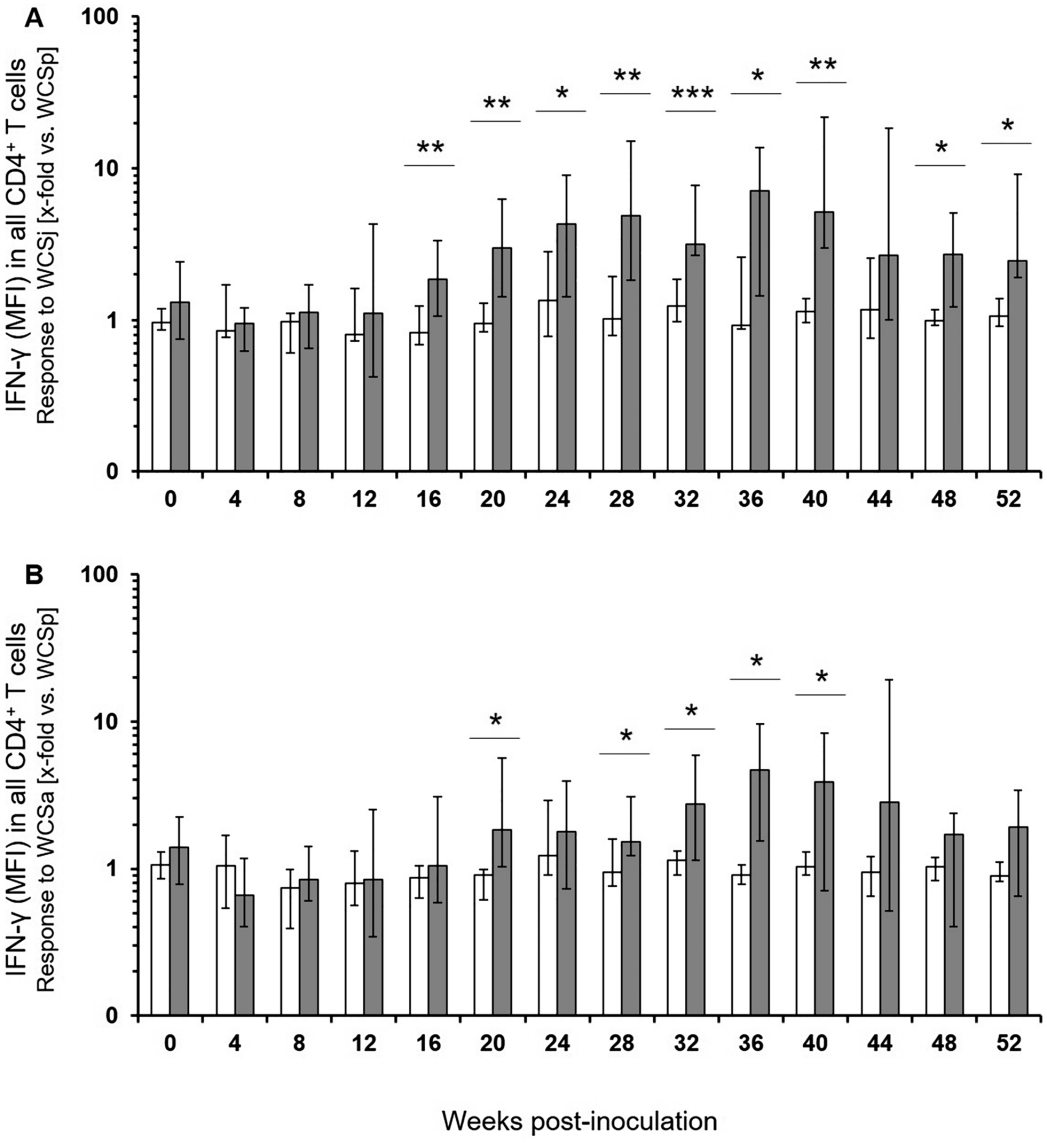
**Mean fluorescence intensity of the IFN-γ signal in CD4**^**+**^** T cells of control group and MAP-infected calves after stimulation with mycobacterial antigens in vitro.** Cells were stimulated with WCSj (**A**) and WCSa (**B**). Data were normalised to values obtained after WCSp stimulation. For further details see legend to Figure 5.

When normalized to the response to a distantly related antigen (PPDp), median responses to PPDj of CD4^+^ T cells from MAP-infected calves and control calves were similar up to 12 wpi (Figure 5A). A significantly higher response of MAP-infected calves was detected at 24, 32 and 40 wpi. Similar results were obtained after stimulation with PPDa (Figure 5B), with cells from MAP-infected calves responding significantly stronger at 12, 32 and 40 wpi. MFI of IFN-γ signals in CD4^+^ T cells after in vitro-stimulation with WCSj antigen allowed for a more consistent differentiation between animal groups (Figure 6A). This difference became significant as early as at 16 wpi and remained significantly different until the end of the sampling period (except week 44 wpi). Responses to stimulation with WCSa were less prominent as compared to WCSj but also were significantly different between cells from control and infected calves at 20, 28, 32, 36 and 40 wpi (Figure 6B).

### Individual responsiveness of MAP-infected and control calves in the FCA

**Figure 7:**
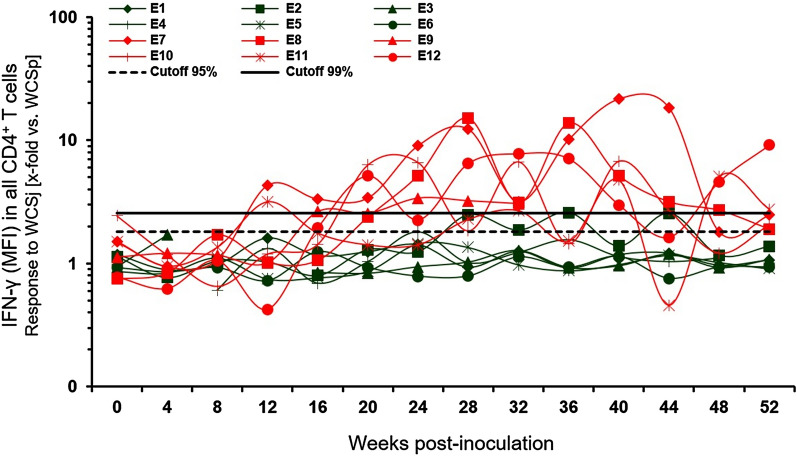
**Relative mean fluorescence intensities of the IFN-γ signal in WCSj-stimulated CD4**^**+**^** cells of each individual calf.** Data were normalized to values from WCSp stimulated PBMC. Green symbols indicate calves of the control group, red symbols MAP-infected calves. The cut-offs were set according to the 95% and 99% quantiles of the values obtained from control calves over time. The values under the 95% quantile were accepted as negative. The values between two cut-offs were interpreted as inconclusive and the values above the 99% quantile as positive.

Group difference are of limited diagnostic value at individual animal level, which led us to analyse data also individually over time taking the 95 and 99% quartiles of all values from the control group as cut-off for inconclusive and positive results, respectively. PPDj and PPDa were similarly capable of stimulating PBMC from MAP-infected animals. In total, 40.0% and 38.2% of samples taken from MAP-infected calves from 16 wpi onwards were graded positive or inconclusive after stimulation of PBMC with PPDj and PPDa, respectively (data not shown). When WCSj was applied for stimulation in vitro instead (Figure 7), one calf (E10) tested inconclusive prior to inoculation. Responses of PBMC from all calves were negative at 4 and 8 wpi. Two infected calves (E7, E11) tested positive as early as 12 wpi. While animal E7 remained positive for the subsequent time points, other inoculated calves showed variable responses mostly in the positive range. Only one control calf (E2) tested positive or inconclusive in several wpi. Overall, stimulation with WCSj led to positive or inconclusive assessment of 74.6% of all samples taken from MAP-infected calves from 16 wpi onwards. At 28, 32, 40 and 52 wpi, all infected calves tested positive or inconclusive. Stimulation of PBMC with WCSa led to lower responses than stimulation with WCSj but the dynamic of the animals’ cell reactivities was similar (Additional file 4). Using WCSa as stimulating agent, 52.7% of all samples taken from MAP-inoculated animals from 16 wpi onwards were graded positive or inconclusive.

### Sensitivity and specificity of CMI tests in the longitudinal studies

**Figure 8:**
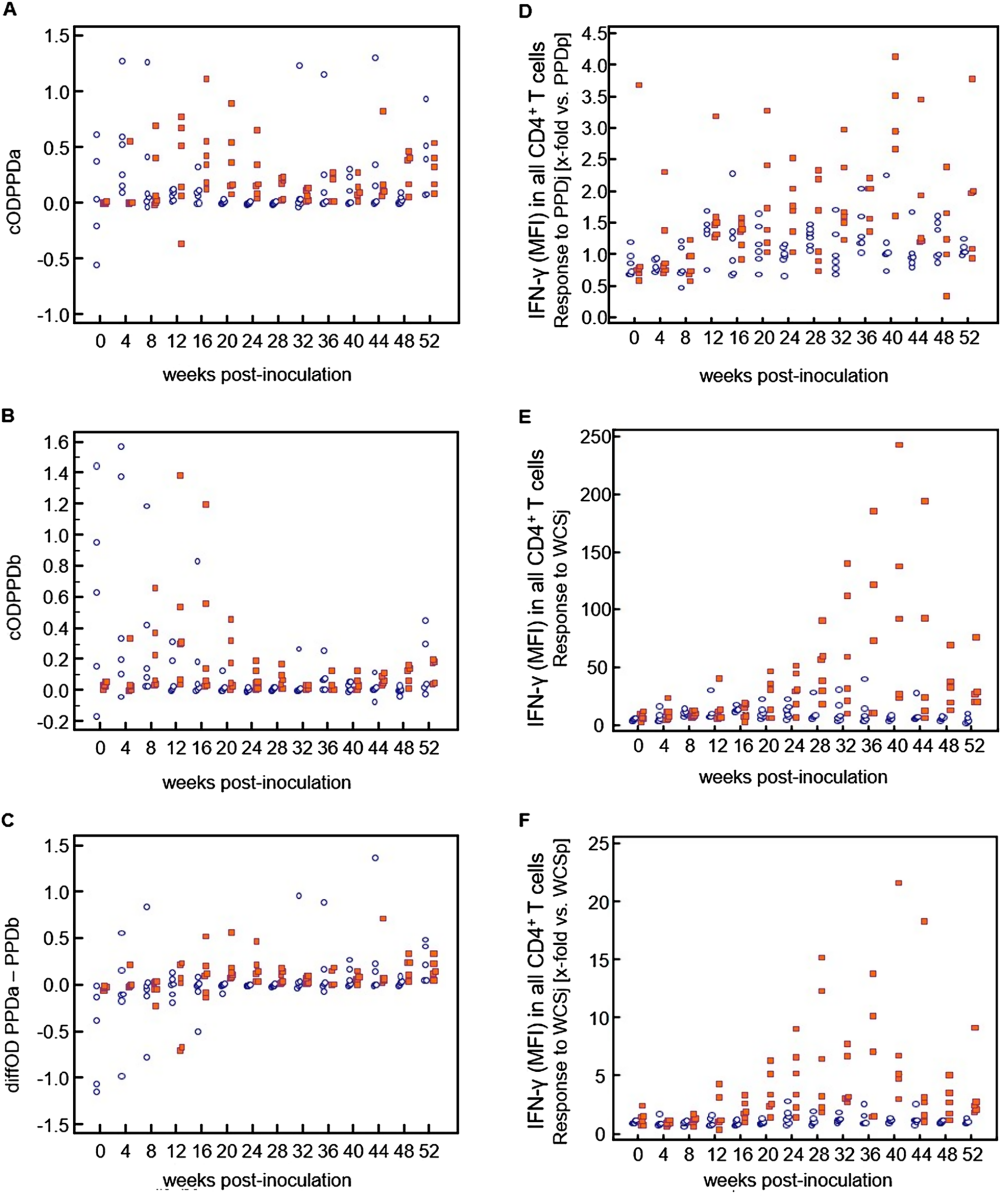
**IFN-γ response detected by the IGRA (A-C) and the FCA (D-F) in calves experimentally infected with MAP (red) compared with the control group (blue).** **A** corrected OD values after stimulation with PPDa; **B** corrected OD values after stimulation with PPDb; **C** Difference in OD values between PPDa- and PPDb-stimulated PBMC; **D** IFN-γ (MFI) in all CD4^+^ T cells, response to PPDj [x-fold vs. PPDp]; **E** IFN-γ (MFI) in all CD4^+^ T cells, response to WCSj; **F** IFN-γ (MFI) in all CD4^+^ T cells, response to WCSj [x-fold vs. WCSp].

In order to assess the relative applicability of the different technical approaches deployed in this study to detect MAP infection in young cattle in a statistical manner, data were first plotted as a comparison of the groups (control vs. MAP-infected) over time to recognize the distribution of the measured values per group (Figure 8). By IGRA, high cOD values were detected up to 16 wpi in control calves after in vitro-stimulation with PPDa or PPDb suggestive of a non-specific response to the antigen stimulations. High IFN-γ values were also occasionally observed in control calves at later time points.

As described above, CD4^+^ T cells from MAP-infected calves produced higher amounts of IFN-γ upon in vitro-stimulation with WCSj and PPDj than in response to WCSa, WCSp, PPDa and PPDp. A receiver-operating characteristic (ROC) curve analysis was carried out separately for each selected parameter. Initially, the values of all time points were analyzed. In a second round of analysis, only the values from 20 wpi were included. For all tested parameters, the area under the curve (AUC) was larger when only values from 20 wpi onwards were included than when all values were included (Table 3).

**Table 3:** **ROC-Analysis of the IGRA- and the FCA data; the AUC for the all measured values over the time and for the period ≥ 20 weeks post-inoculation (wpi)**

Method	Parameter	AUC			
		All time points		Time points at ≥ 20 wpi	
			95% CI*		95% CI*
IGRA	cOD_PPDa_	0.704	0.627–0.773	0.781	0.688–0.857
	cOD_PPDb_	0.663	0.584–0.736	0.762	0.667–0.841
	diffOD _PPDa–PPDb_	0.716	0.639–0.784	0.765	0.671–0.844
FCA [MFI of CD4^+^/IFN-γ^+^ T cells]	Response to PPDj [x-fold vs. PPDp]	0.737	0.661–0.803	0.798	0.707–0.871
	Response to WCSj	0.808	0.738–0.866	0.903	0.828–0.952
	Response to WCSj [x-fold vs. WCSp]	0.848	0.782–0.900	0.954	0.893–0.985

^*^CI: confidence interval.

**Figure 9:**
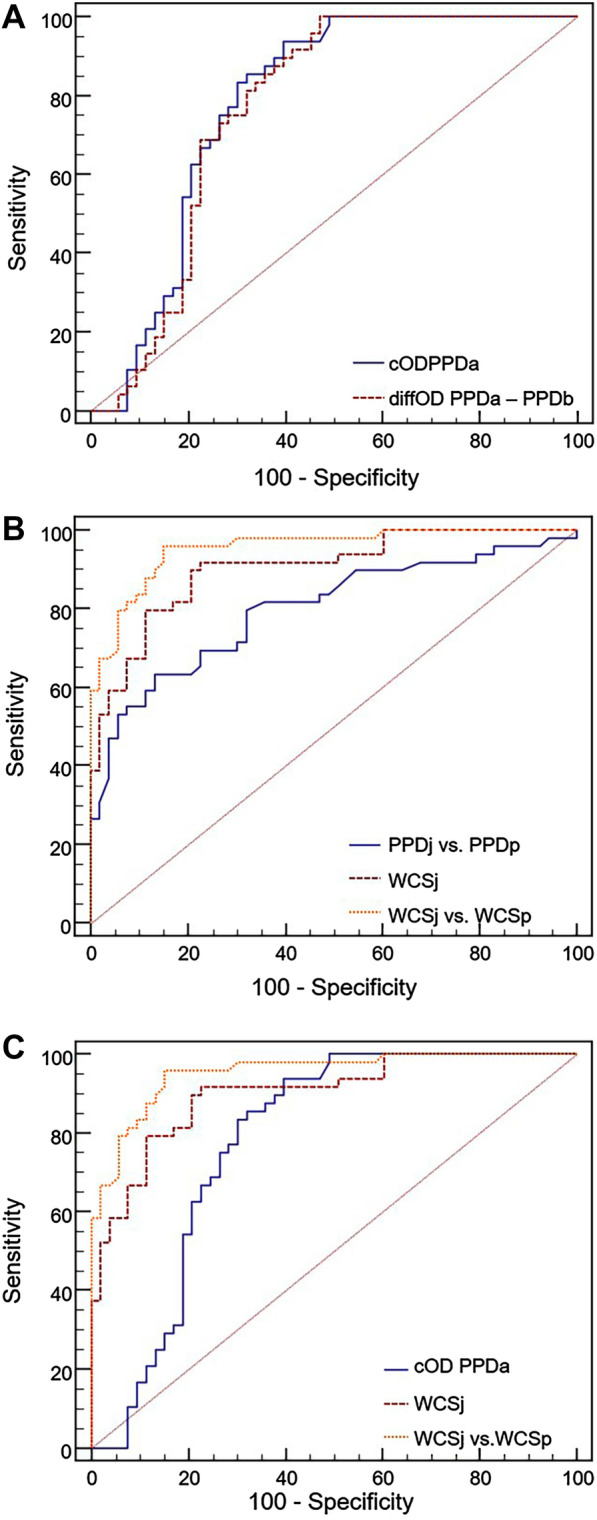
**ROC curves obtained by different methods and parameters to assess the antigen-specific cellular immune response of MAP-infected calves.** Only values from 20 wpi onwards were considered. **A** IGRA data using the evaluation parameters cOD_PPDa_ und diffOD_PPDa – PPDb_. **B** FCA data using the evaluation parameters “IFN-γ (MFI) in all CD4 + T cells, response to PPDj [x-fold vs. PPDp]” (designated “PPDj vs. PPDp”), “IFN-γ (MFI) in all CD4 + T cells, response to WCSj” (designated “WCSj”) and “IFN-γ (MFI) in all CD4 + T cells, response to WCSj [x-fold vs. WCSp]” (designated “WCSj vs. WCSp”). **C** IGRA data (cOD_PPDa_) and FCA data using the evaluation parameters “IFN-γ (MFI) in all CD4 + T cells, response to WCSj” (designated “WCSj”) and “IFN-γ (MFI) in all CD4 + T cells, response to WCSj [x-fold vs. WCSp]” (designated “WCSj vs. WCSp”).

If only values from 20 wpi onwards were considered, a good test performance was achieved with the IGRA using the evaluation parameters cOD_PPDa_ and diffOD_PPDa–PPDb_. The performance of the two parameters did not differ significantly from one another (Figure 9A). For FCA, the use of the evaluation parameters “IFN-γ (MFI) in all CD4^+^ T cells, response to WCSj” and “IFN-γ (MFI) in all CD4^+^ T cells, response to WCSj [x-fold vs. WCSp]” resulted in better test performance than the parameter “IFN-γ (MFI) in all CD4^+^ T cells, response to PPDj [x-fold vs. PPDp]”. The parameter “IFN-γ (MFI) in all CD4^+^ T cells, response to WCSj [x-fold vs. WCSp]” performed best (Figure 9B). Parameters “IFN-γ (MFI) in all CD4 + T cells, response to WCSj” and “IFN-γ (MFI) in all CD4 + T cells, response to WCSj [x-fold vs. WCSp]” of the FCA displayed a clearly better performance than the IGRA (Figure 9C).

Cut-off values were defined for four different evaluation parameters to differentiate between positive and negative assay results. For this purpose, cut-off values were selected to optimally balance between sensitivity (Se) and specificity (Sp; Table 4). If the cut-off 0.1, prescribed by the manufacturer to assess the bovine tuberculosis status of the tested animal, was applied to all values from 20 wpi and onwards, the sensitivity of the IGRA was lower but the specificity was higher than if optimized cut-off values were applied. Of note, parameters “IFN-γ (MFI) in all CD4^+^ T cells, response to WCSj” and “IFN-γ (MFI) in all CD4^+^ T cells, response to WCSj [x-fold vs. WCSp]” of the FCA were characterized by a higher Se and Sp than the any of the interpretations of the IGRA.

**Table 4:** **Test characteristics of selected evaluation parameters from the IGRA and the FCA for the period ≥ 20 weeks post-inoculation (wpi)**

Parameter	Cut-off	Sensitivity (%)	Specificity (%)
IGRA–cOD_PPDa_	0.086^1^	70.8	73.6
IGRA–diffOD_PPDa–PPDb_	0.045^1^	72.9	73.6
IGRA–cOD_PPDa_	0.102^2^	66.7	77.4
IGRA–diffOD_PPDa–PPDb_	0.102^2^	47.9	79.2
FCA–MFI of CD4^+^/IFN-γ^+^ T cells, response to WCSj	18.5	79.6	88.7
FCA–MFI of CD4^+^/IFN-γ^+^ T cells, response to WCSj [x-fold vs. WCSp]	1.53	87.8	86.8

^1^Optimized cut-off.

^2^The cut-off 0.1 is prescribed by the manufacturer to assess the bovine tuberculosis status.

## Discussion

Even more than a century after its first description, diagnosis and control of Ptb remains challenging [2]. An early pro-inflammatory response to MAP infection suggests that testing for the key effector cytokine IFN-γ may detect animals much earlier than serum ELISA or faecal culture [5]. Results presented here imply that the performance of a flow cytometry-based quantification of IFN-γ production by CD4^+^ lymphocytes, a method that was suggested previously [19], can be further improved by using more potent antigen preparations for in vitro stimulation and by applying an optimized strategy of data analysis and normalization to detect MAP-infected cattle even at an age of 20 weeks.

The variability of potency and lack of production consistency in PPD’s is well documented [58] and the concentration of PPDj applied in IFN-γ tests varies considerably between laboratories [59]. Recombinant proteins were considered an alternative for CMI-based diagnostic tests [31]. However, due to individual variations of the responses elicited by infected animals to the same antigenic stimuli, a cocktail of antigens may be required to detect as many affected animals as possible, i.e., increase the sensitivity of the test [14]. In most experimental studies with MAP-infected calves, complex antigen mixtures like WCSj and/or PPDj were deployed as test antigen to detect CMI responses [13, 19, 60]. Using WCS and PPD antigens, derived from MAP, *M. avium* ssp. *avium*, and *M. phlei*, in parallel, the response of MAP-infected cattle to the WCSj antigen preparation used herein was the strongest. Although the antigenic characteristic of PPDj was reported in literature, information is scarce on the proteomic content of WCSj. PPDs derived from *M.* *avium* ssp. *avium*, MAP and *M. bovis* contain 156, 95 and 132 proteins respectively [36]. Investigations on the composition of the PPDs and WCSs and determining the optimal concentration of the respective preparations to achieve a superior signal-to-noise ratio was beyond the manageability of the presented study. Further efforts into these directions seem to bear the potential to further improve the test outcome.

Identification of cattle with Ptb by IGRA has followed different approaches, varying in the type of samples and test antigens, concentration of the antigens used and the interpretation of the test results [55]. Reportedly, the diagnostic performance of IGRA is better than the serum ELISA to detect MAP-specific antibodies for animals at 1–2 years of age, and the serum and the milk ELISA for animals at 2–3 years of age, i.e., immediately prior to development of clinical disease [16]. Nevertheless, IGRA has not become established to detect Ptb due to inconsistent reports on its diagnostic predictive value [61, 62]. Indeed, even though handling, incubation period, stimulation dosages, animal age and interpretation of the test results were kept as constant as possible throughout the current study, two out of seven cows, which shed MAP bacteria in their faeces and were serologically MAP-positive, reacted test-negative and a sample of one additional cow yielded inconclusive results. Samples from control group calves showed high cOD values after stimulation with PPDa or PPDb up to 16 wpi, indicative of unspecific stimulation. At later time points, high cOD values also occasionally occurred in control animals. Cut-offs used for test interpretation differ between laboratories [63]. Optimization of the test interpretation has been tried before by variation of cut-off values [55] or by application of different interpretation schemes and algorithms [64]. Repeated sampling over several months can unveil significant differences between MAP-infected and control calves [65]. It has been suggested that refinements in test interpretation and repeated testing may resolve the shortcomings of the IGRA, but high costs of handling and the long time between sampling and test interpretation have prevented broader application in the field that far [14].

Long-term in vitro culture of PBMC with MAP antigens resulted in a change in percentages of the affected cell populations [66]. Incubation with antigens for 6 days not only allows development of mature antigen-presenting cells but also the differentiation of these cells which is a valid approach to measure recall responses [19]. In the present study, the method described by Waters et al. [19] was modified as such that in vitro antigen stimulation of PBMC cultures was conducted for six instead of 7 days and mitogenic activation of the PBMC culture was shortened to 4 h. Minor changes (i.e., incubation time between the staining steps, fluorescent dyes used) during immunolabeling were also introduced. Orientating experiments with PBMC from adult cattle for the establishment of the method showed that a 6 day incubation results in a reliable differentiation of the T cell populations (lymphocytes and lymphoblasts), in particular, CD4^+^ and CD8^+^ IFN-γ producing T cells and in a substantial signal for the detection of IFN-γ in CD4^+^ lymphoblasts by flow cytometry. Experimental studies suggest that the main source of IFN-γ during MAP infection of cattle is the CD4^+^ T cell subpopulation [13, 19]. However, the lymphoid system of cattle contains a large number of γδ T cells, which is an obstacle when testing young ruminants in which up to 60% of the lymphocytes have γδ T cell receptors [67]. The dominance of γδ T cells is the apparent explanation for the low number of CD4^+^ T cells detectable in 6 day-old PBMC cultures of the calves in the longitudinal studies. Of note, PBMC cultures of infected animals supplemented with MAP-antigen comprised elevated total numbers of CD4^+^ T cells. It appears that the extended incubation period of the FCA compared to the IGRA allowed antigen-specific lymphocytes not only to become activated and to produce IFN-γ but also to undergo significant numerical expansion by proliferation. In terms of diagnostic sensitivity, these two phenomena seem to act in synergy, which is particularly beneficial when it comes to apply this approach to samples of young animals. ROC analyses conducted in this study comparing the diagnostic performances of IGRA and FCA support this assumption. Even though a laboratory workflow lasting for a week is disadvantageous for the field applicability of the method, the superior performance at calves’ age of the FCA evaluated herein justifies its further consideration for improving and simplification. One aspect to be considered is the establishment of decision rules for the interpretation of inconclusive results if the test is to be applied at the level of individual animals. Individual data presented in Figure 7 indicate that samples from animals of both groups occasionally yielded inconclusive results. Repeated testing of animals or combination with other test, not different from what is currently applied in Ptb control programmes, may mitigate this problem.

The present study conducted a comparative analysis to develop the most suitable analysis strategy based on data obtained from adult cattle. The MFI reflects the average amount of fluorescence at the level of individual cells, whereas the percentage of responding cells takes the whole T cell population into account. When testing adult cattle naturally infected with MAP, MFI values of the IFN-γ signal in all CD4^+^ T cells (lymphocytes and lymphoblasts) significantly differed between negative and MAP-infected cattle after stimulation with PPDa, PPDj and WCSj. The antigen-specific response became even more obvious when the same parameter was determined for lymphoblasts only. This was also observed for the percentage of double positive cells (CD4^+^/IFN-γ^+^) in our study, similar to what has been reported for cattle subclinical infected with MAP [19, 21, 37]. Different from sheep and goats and similar to other studies [13, 66, 68, 69] no reactivity of CD8^+^ cells to mycobacterial antigens could be detected by the protocol applied herein. Findings from the pilot study led us to assume that CD4^+^ lymphoblasts are the most suitable target cell population for IFN-γ quantitation to detect MAP-positive adult cattle. However, if numbers of detectable CD4^+^/IFN-γ^+^ cells are considerably low, as in PBMC cultures from calves, it proved suitable to analyse the lymphocyte and lymphoblast populations together, instead.

Antigen-specific cellular immune responses detected in MAP-infected ruminants vary between studies. Experimental infection of neonatal lambs with MAP yielded significantly higher quantities of CD8^+^ T cells [70]. In naturally infected sheep, the number of the CD4^+^ T cells remain to be unchanged during the different disease stages but an increased number of CD8^+^ T cells was observed in paucibacillary sheep [71]. Navarro et al. reported decreased numbers of CD4^+^ T cells and increased numbers of CD8^+^ T cells in mesenteric lymph nodes in MAP-infected goats [72]. It remains to be determined, whether these findings truly reflect differences between host species or can rather be attributed to differences in the study design, the sampling matrix or the stage of disease. Nevertheless, it has to be assessed, how the FCA approach needs to be amended to be successfully applied to other ruminants.

The present study aimed at monitoring the dynamics of the MAP-specific CMI in experimentally infected calves in order to define the earliest time point of the infection at which reliable diagnosis is achievable. In a similar approach, Waters, Miller, Palmer, Stabel, Jones, Koistinen, Steadham, Hamilton, Davis and Bannantine [19] successfully detected antigen-specific CMI in PBMC of infected calves at 194 days post-inoculation (i.e., at about 25–26 wpi). Similarly, and later corroborated by others [13, 19, 38, 73], antigen-specific responses in PBMC cultures of MAP-infected animals were detected 6 months after inoculation while the results of IFN-γ assays and ELISAs were variable [13]. The results of the present study showed that the MFI of IFN-γ signals in CD4^+^ cells after in vitro stimulation showed the most consistent differentiation between animal groups, significant as early as from 16 wpi onwards. Considering that the model of T_H_1/T_H_2 response in MAP infections is still to be unravelled [66], long term investigations on experimentally infected calves beyond 52 wpi are required to understand how antigen-specific responses of the bovine hosts develop in the subsequent stage of subclinical infection. Pending optimisation to promote the usability of this test in the field notwithstanding, the FCA has proven its potential to overcome some limitations of the current diagnostic tests [74] and may be particularly useful, e.g. for screening young animals destined for breeding stock [14].

### Supplementary Information

**Additional file 1. Serology and faecal examinations of adult cattle of the pilot study**.

**Additional file 2. Interferon gamma release assay of adult cattle of the pilot study**.

**Additional file 3. Portion and Interferon gamma production by PBMC from adult cattle determined by FCA**. Relative portion and IFN-γ production by PBMC from MAP-unexposed and MAP-infected cows after in vitro stimulation with mycobacterial antigens independent of lymphocyte subset. Data are median (+ / - max / min) of the values with duplicates from MAP-unexposed (*n* = 5) white bars] and MAP-infected (*n* = 7) animals grey bars], normalised to values obtained after stimulation with a respective (i.e., PPD and WCS, respectively) *M phlei* preparation. Asterisks above each condition indicate significant differences between MAP-unexposed and MAP-infected cows (Student’s *t*-test; *p* ≤ 001 (**), or *p* ≤ 005 (*)). MFI: Mean fluorescence intensity.

**Additional file 4. Interferon gamma production by CD4 cells from calves determined by FCA.** Relative mean fluorescence intensities of the IFN-γ signal in WCSa-stimulated CD4^+^ cells of each individual calf. Data were normalized to values from WCSp stimulated PBMC Green symbols indicate calves of the control group, red symbols MAP-infected calves. The cut-offs were set according to the 95% and 99% quantiles of the values obtained from control calves over time. The values under the 95% quantile were accepted as negative. The values between two cut-offs were interpreted as inconclusive and the values above the 99% quantile as positive.

## Data Availability

The raw data supporting the conclusions of this article are available from the corresponding author on reasonable request.
