# Supplementary material for: Interferon-gamma producing CD4+ T cells quantified by flow cytometry as early markers for Mycobacterium avium ssp. paratuberculosis infection in cattle
Source: Vet Res. 2024 May 31;55:69. doi: 10.1186/s13567-024-01324-8 (PMC11143577; doi:10.1186/s13567-024-01324-8)
Supplement: Supplementary file 1 — Additional file 1. Serology and faecal examinations of adult cattle of the pilot study. [file 13567_2024_1324_MOESM1_ESM.docx]

**Additional file 1** **Adult cattle of the pilot study.** Results of serology and faecal examinations are indicated as not tested (n.t.), test-negative (-), inconclusive (?) and positive (+).

|  |  |  | Serology | |  | Faecal examination | |
| --- | --- | --- | --- | --- | --- | --- | --- |
| Cattle No. | Age (year) |  | ^1^Svanovir® ELISA | ^2^Pourquier® ELISA |  | ^3^PCR | ^4^Culture |
| Neg-1 | 3.2 |  | **-** | **-** |  | **-** | **-** |
| Neg-2 | 3.2 |  | **-** | **-** |  | **-** | **-** |
| Neg-3 | 3.3 |  | **-** | **-** |  | **-** | **-** |
| Neg-4 | 4.9 |  | **n.t.** | **-** |  | **-** | **-** |
| Neg-5 | 5.0 |  | **n.t.** | **-** |  | **-** | **-** |
| Pos-1 | 3.3 |  | **?** | **+** |  | **+** | **+** |
| Pos-2 | 3.2 |  | **?** | **+** |  | **+** | **+** |
| Pos-3 | 3.0 |  | **?** | **+** |  | **+** | **+** |
| Pos-4 | 4.6 |  | **-** | **+** |  | **+** | **-** |
| Pos-5 | 5.5 |  | **-** | **+** |  | **+** | **+** |
| Pos-6 | 4.1 |  | **+** | **+** |  | **+** | **+** |
| Pos-7 | 4.9 |  | **+** | **+** |  | **+** | **+** |

^1^Svanovir® Para-TB-Ab-ELISA, Svanova, Sweden; ^2^ Pourquier® ELISA-Paratuberculosis screening, Inst. Pourquier, France; ^3^Real Time Duplex PCR (IS*Mav2* and F*57*) and IS*900*-nested PCR [48], ^4^ Herold Egg Yolk Med. (HEYM) >12 weeks incubation.
