# Supplementary material for: Interferon-gamma producing CD4+ T cells quantified by flow cytometry as early markers for Mycobacterium avium ssp. paratuberculosis infection in cattle
Source: Vet Res. 2024 May 31;55:69. doi: 10.1186/s13567-024-01324-8 (PMC11143577; doi:10.1186/s13567-024-01324-8)
Supplement: Supplementary file 2 — Additional file 2. Interferon gamma release assay of adult cattle of the pilot study. [file 13567_2024_1324_MOESM2_ESM.docx]

**Additional file 2 IFN-γ release in antigen stimulated whole blood cell cultures of cows (IGRA)**

| Cattle No. | Age (year) | Calculated OD_[450-620nm]_ | | Test result |
| --- | --- | --- | --- | --- |
|  |  | [PPDa]-[Nil*] | [PPDa]-[PPDb] |  |
| Neg-1 | 3.2 | 0.06 | 0.04 | - |
| Neg-2 | 3.2 | 0.01 | 0.00 | - |
| Neg-3 | 3.3 | 0.15 | 0.08 | ? |
| Neg-4 | 4.9 | 0.03 | 0.03 | - |
| Neg-5 | 5.0 | 0.02 | 0.02 | - |
| Pos-1 | 3.3 | 0.26 | 0.20 | + |
| Pos-2 | 3.2 | 0.29 | 0.23 | + |
| Pos-3 | 3.0 | 0.05 | 0.03 | - |
| Pos-4 | 4.6 | 0.22 | 0.15 | + |
| Pos-5 | 5.5 | 1.21 | 0.93 | + |
| Pos-6 | 4.1 | 0.01 | 0.00 | - |
| Pos-7 | 4.9 | 0.11 | 0.07 | ? |

*Nil: Samples supplemented with PBS; for interpretation of tests results see Materials and methods.
