# Supplementary figures and images for: Interferon-gamma producing CD4+ T cells quantified by flow cytometry as early markers for Mycobacterium avium ssp. paratuberculosis infection in cattle
Source: Vet Res. 2024 May 31;55:69. doi: 10.1186/s13567-024-01324-8 (PMC11143577; doi:10.1186/s13567-024-01324-8)

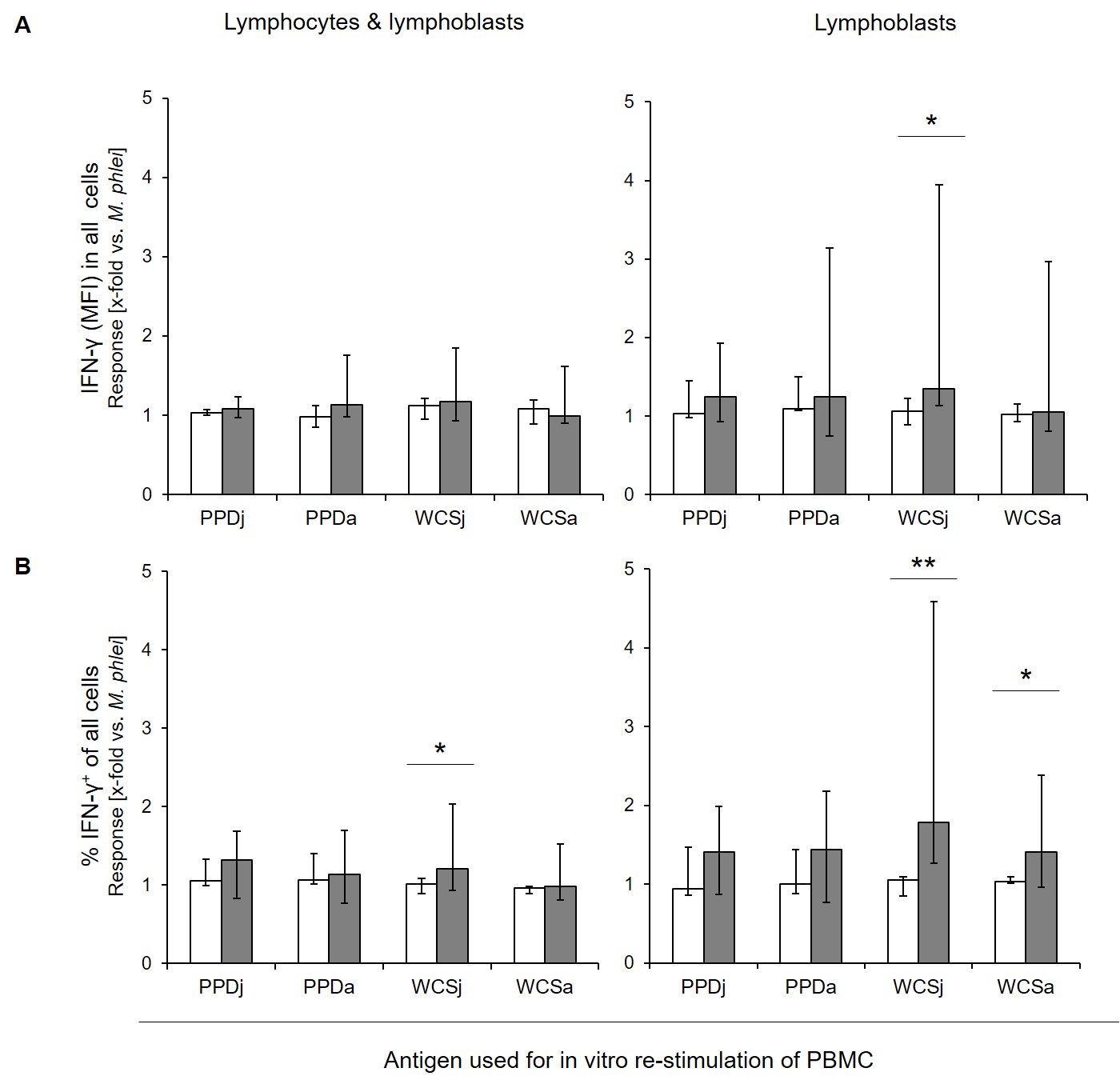

Supplement: Supplementary file 3 — Additional file 3. Portion and Interferon gamma production by PBMC from adult cattle determined by FCA. Relative portion and IFN-γ production by PBMC from MAP-unexposed and MAP-infected cows after in vitro stimulation with mycobacterial antigens independent of lymphocyte subset. Data are median (+ / - max / min) of the values with duplicates from MAP-unexposed (n = 5) white bars] and MAP-infected (n = 7) animals grey bars], normalised to values obtained after stimulation with a respective (i.e., PPD and WCS, respectively) M phlei preparation. Asterisks above each condition indicate significant differences between MAP-unexposed and MAP-infected cows (Student’s t-test; p ≤ 001 (**), or p ≤ 005 (*)). MFI: Mean fluorescence intensity. [file 13567_2024_1324_MOESM3_ESM.jpg]

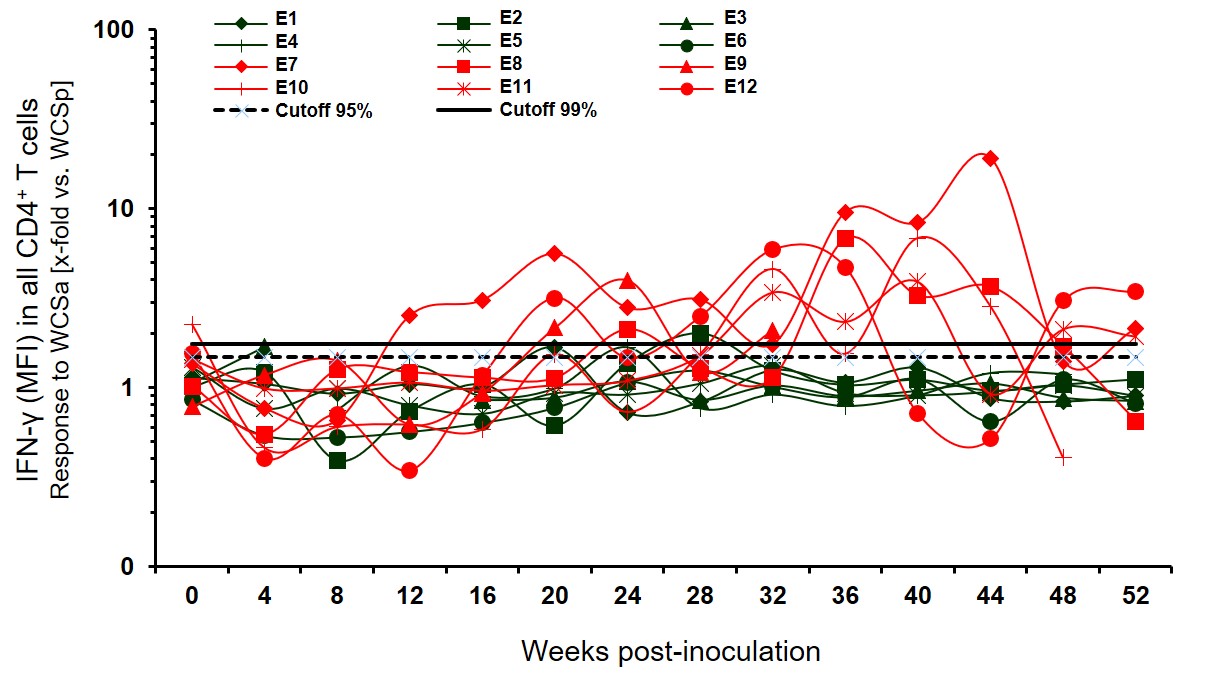

Supplement: Supplementary file 4 — Additional file 4. Interferon gamma production by CD4 cells from calves determined by FCA. Relative mean fluorescence intensities of the IFN-γ signal in WCSa-stimulated CD4+ cells of each individual calf. Data were normalized to values from WCSp stimulated PBMC Green symbols indicate calves of the control group, red symbols MAP-infected calves. The cut-offs were set according to the 95% and 99% quantiles of the values obtained from control calves over time. The values under the 95% quantile were accepted as negative. The values between two cut-offs were interpreted as inconclusive and the values above the 99% quantile as positive. [file 13567_2024_1324_MOESM4_ESM.jpg]
